# Hemoglobin is associated with BMDs and risk of the 10-year probability of fractures in patients with type 2 diabetes mellitus

**DOI:** 10.3389/fendo.2024.1305713

**Published:** 2024-01-22

**Authors:** Ren-xuan Li, Na Xu, Yu-ning Guo, Yan Wang, Yan-wei Liang, Xiao-lian Zhou, Wen-tong Jiang, Jian-xia Wei, Xin-yuan Zhang, Li-na Zhou, Lei Zhu, Yan-man Zhou, Jin Xu

**Affiliations:** ^1^ Department of Endocrinology, Shandong Provincial Hospital, Shandong University, Jinan, Shandong, China; ^2^ Key Laboratory of Endocrine Glucose & Lipids Metabolism and Brain Aging, Ministry of Education; Department of Endocrinology, Shandong Provincial Hospital Affiliated to Shandong First Medical University, Jinan, Shandong, China; ^3^ Department of Endocrinology, Shandong Clinical Research Center of Diabetes and Metabolic Diseases, Jinan, Shandong, China; ^4^ Department of Endocrinology, Shandong Institute of Endocrine and Metabolic Diseases, Jinan, Shandong, China; ^5^ Department of Endocrinology, “Chuangxin China” Innovation Base of Stem Cell and Gene Therapy for Endocrine Metabolic Diseases, Jinan, Shandong, China; ^6^ Department of Endocrinology, Shandong Engineering Laboratory of Prevention and Control for Endocrine and Metabolic Diseases, Jinan, Shandong, China; ^7^ Department of Endocrinology, Shandong Engineering Research Center of Stem Cell and Gene Therapy for Endocrine and Metabolic Diseases, Jinan, Shandong, China; ^8^ Department of First Clinical Medical College, Shandong University of Traditional Chinese Medicine, Jinan, Shandong, China; ^9^ Department of Endocrinology, Shandong Provincial Third Hospital, Jinan, Shandong, China; ^10^ Department of Nephrology, Shandong Provincial Hospital, Shandong University, Jinan, Shandong, China; ^11^ Department of Nephrology, Shandong Provincial Hospital Affiliated to Shandong First Medical University, Jinan, Shandong, China

**Keywords:** hemoglobin, osteoporosis, type 2 diabetes mellitus, bone mineral density, fracture risk

## Abstract

**Purpose:**

This study aimed to investigate the associations between hemoglobin (HGB) levels and bone mineral density (BMD) and fracture risk in type 2 diabetes mellitus(T2DM) population of different ages.

**Method:**

This cross-sectional study included 641 patients with T2DM (57.9% males). BMD of the femoral neck (FN), total hip (TH), and lumbar spine (LS) were measured using dual-energy X-ray absorptiometry. The 10-year probability of fracture was assessed using a fracture risk assessment tool (FRAX). HGB and other biochemical indices were measured in a certified laboratory at our hospital. Statistical analysis was performed using SPSS 26.0 and R language (R version 4.1.0). Generalized additive models (GAMs) were used to identify the associations between HGB and BMD and fracture risk.

**Results:**

Patients with osteoporosis have lower HGB levels than the non-osteoporotic population and lower FN BMD in patients with anemia than in the non-anemic population. In patients with T2DM, there was sex- and age-related variability in the correlation between HGB levels and BMDs and fracture risk. In older men, HGB level was an independent determinant of BMD and was positively correlated with FN and TH BMD. In non-older women, HGB level was an independent determinant of BMD and fracture risk, positively associated with BMDs and negatively associated with 10-year probability of fracture risk. GAMs revealed a positive linear association between HGB level and BMDs in non-older female patients but not in older male patients.

**Conclusion:**

Our study provides a new perspective on the association of HGB level and BMDs with fracture risk. Relatively high HGB levels are a protective factor for bone quality in patients with T2DM. However, the bone-protective effect of HGB is influenced by age and sex and persists only in older men and non-older women with T2DM.

## 1 Introduction

Osteoporosis is characterized by decreased bone density, resulting in weaker and more fragile bones (1). In China, osteoporotic mortality and morbidity have been on the rise (2). There has been increased diabetes prevalence worldwide every year (3). Osteoporosis is associated with diabetes mellitus (4), and the risk of osteoporosis increases for patients with diabetes mellitus (5). It has been reported that patients with diabetes mellitus are at an increased risk of nonvertebral fracture and osteoporotic vertebral fractures, particularly in Asian populations (6–8).

A global anemia prevalence of 32.9% was recorded by the World Health Organization (WHO) in 2010 (9). Previous studies have shown a rapid increase in anemia prevalence after the age of 50 years, approaching 20% in those aged 85 or older (10). However, anemia incidence in patients with T2DM is much higher than that in the general population (11). Anemia is defined as a decrease in red blood cell production, abnormal red blood cell morphology, or an inadequate amount of hemoglobin in red blood cells, leading to insufficient oxygen and nutrient supply to the body (12, 13). Both inhibitory and enhancing effects of hypoxia on osteocytic differentiation have been observed in humans (14–17). A previous clinical study revealed a correlation between low hemoglobin (HGB) levels and an elevated risk of osteoporosis in the adult population (18). In a cross-sectional study, it was found that HGB levels were associated with the presence of osteoporosis in men with T2DM, particularly in patients aged 50 and above (19).

It is well known that the risk factors for osteoporosis vary according to sex and age. Previous studies indicated that the prevalence of osteoporosis increases with advancing age (20). According to one study, osteoporosis prevalence increased to 32.0% among those over 65 years of age, as opposed to 19.2% in those over 50 years of age (21). Research has shown that after the age of 50, osteoporosis prevalence in men is approximately 13%, significantly lower compared to women of the same age group, where the prevalence is around 40% (22). In particular, the prevalence rate of osteoporosis in postmenopausal women in China is as high as 60% (23).

Previous studies have indicated that anemia is associated with osteoporosis in patients with T2DM, regardless of sex and without age stratification (24). Several studies have investigated the association between HGB levels and BMD in the general population. Our study stratified the analysis according to age and sex in the type 2 diabetes mellitus (T2DM) population to investigate the association between HGB levels and BMD.

## 2 Methods

### 2.1 Study participants

This study used a cross-sectional design. The study population consisted of patients with T2DM hospitalized at the Department of Endocrine and Metabolic Diseases, Shandong First Medical University Affiliated Provincial Hospital, from October 2021 to June 2023. The inclusion criteria were age ≥18 years and the presence of T2DM. The exclusion criteria were as follows: 1) patients with type 1 diabetes mellitus or diabetes of any other type and acute complications of diabetes mellitus; 2) other chronic disorders that affect mineral metabolism, such as Paget disease of the bones, significant liver or kidney disease, as well as untreated thyroid disorders and Cushing’s syndrome; 3) a history of malignancy; and 4) taking drugs that may affect bone metabolism and anemia, such as glucocorticoids, sex hormone therapy, thiazolidinedione, folic acid, iron supplements, bisphosphonates, Alfacalcidol, Miacalcic, or calcitonin drugs. This study was performed in line with the principles of the Declaration of Helsinki. Approval was granted by the Institutional Review Board of Shandong Provincial Hospital affiliated with Shandong First Medical University (SWYX: NO.2023-284). All participants signed an informed consent.

### 2.2 General data

To gather information, we thoroughly examined each individual’s medical records. The following clinical data were collected: 1) Patient demographics, such as age, sex, height, and weight. 2) Medical history of T2DM, including the time of diagnosis, illness duration, and treatment details. 3) Smoking status, alcohol consumption, and hypertension history. Body mass index (BMI; kg/m^2^) was calculated as weight/height^2^.

### 2.3 Biochemical measurements

Blood samples were collected from the participants after an 8–12 hour fasting period. The collected samples were sent to a certified laboratory at our hospital for analysis. The collected biochemical data included glycated hemoglobin A1C (HbA1c), Alkaline phosphatase (ALP), high-density lipoprotein cholesterol (HDL), low-density lipoprotein cholesterol (LDL), total cholesterol (TC), triglyceride (TG), calcium (Ca), phosphorus (P), fasting blood glucose (FBG), serum uric acid (SUA), serum creatinine (SCR), parathyroid hormone (PTH), N-terminal medium molecular fragment of osteocalcin (N-MID Ost), β-Collagen special sequence (β-Cros), Total type I collagen amino-terminal extension peptide (T-PINP), 25-hydroxyvitamin (25-VIT D), white blood cells (WBC), HGB, lymphocytes (Lymph), monocytes (Mono), neutrophils (Neut). According to WHO hemoglobin cutoffs, anemia was defined as HGB <130 g/L in men and HGB <120 g/L in women.

### 2.4 Bone mineral density measurement and fracture risk assessment

BMD of the femoral neck (FN), total hip (TH) and lumbar spine (LS) were measured using dual-energy X-ray absorptiometry (Hologic Horizon W, USA). According to the WHO criteria, non-osteoporosis was defined as a T-score > -2.5. Osteoporosis was defined as a T-score ≤ -2.5 SD in any of the three sites. The fracture risk was assessed using the China-specific fracture risk assessment tool (FRAX) (https://www.sheffield.ac.uk/FRAX/tool.aspx?country=2) to estimate the 10-year probability of major osteoporotic fractures (MOF) and hip fractures (HF) in each participant.

### 2.5 Statistical analysis

Continuous data are expressed as mean ± SD for normally distributed variables and medians with 25th and 75th percentiles for abnormally distributed variables. Two-sample t-tests were employed to assess differences in means between groups, whereas the Mann-Whitney U test was utilized to examine differences in medians between groups. Categorical variables are presented as numbers and percentages. Pearson’s correlation analysis was performed to investigate the association of HGB levels with BMD, MOF, and HF. Multiple logistic regression analysis was performed to ascertain the association between HGB levels, BMD, and future fracture risk, with adjustment for covariates. We employed a generalized additive model (GAM) to evaluate the non-linear relationship between HGB levels and BMD, MOF and HF. All statistical analyses were conducted using SPSS 26.0, R language (R version 4.1.0), and figures were created using GraphPad Prism 8. P values < 0.05 were considered statistically significant.

## 3 Results

### 3.1 Baseline characteristics

A total of 641 participants (371 males and 270 females) were included in the analysis according to the inclusion and exclusion criteria. **Table 1** shows the general characteristics of participants. The mean age of the participants was 56.03 ± 13.42 years, and the mean HbA1c (%) level was 9.11 ± 2.14; the mean BMD of FN, TH, and LS were 0.76 ± 0.14 g/cm^2^, 0.89 ± 0.14 g/cm^2^, and 0.94 ± 0.15 g/cm^2^, respectively. Participants were divided into two groups according to their BMD T-scores (osteoporotic and non-osteoporotic). Compared to patients without osteoporosis, those with osteoporosis were older, had lower BMIs, longer diabetes duration, lower TG, higher HDL, and lower HGB levels.

**Table 1:** Baseline characteristics of groups stratified by BMD T-Scores.

Variables	Total (n=641)	T-Score > -2.5 (n=550)	T-Score ≤-2.5 ^a^(n=131)	P
Male, n (%)	371(57.9%)	327(64.1%)	44(33.6%)	
Age (years)	56.03±13.42	54.30±13.55	62.90±10.26	<0.001
BMI (kg/m^2^)	25.92±4.00	26.29±4.02	24.26±3.37	<0.001
Diabetes duration (years)	10(2-16)	8(2-15)	11(4-19)	0.008
HbA1c (%)	9.11±2.14	9.17±2.15	8.88±2.07	0.171
ALP (U/L)	76(61.50-92)	73(60-90)	84(67103)	<0.001
TG (mmol/L)	1.43(0.94-2.09)	1.47(1.97-2.13)	1.25(0.92-1.78)	0.037
TC (mmol/L)	4.85±1.32	4.84±1.30	4.91±1.43	0.602
HDL (mmol/L)	1.19(1.00-1.44)	1.17(0.98-1.42)	1.29(1.08-1.51)	0.002
LDL (mmol/L)	3.02(2.36-3.67)	3.02(2.42-3.67)	2.02(2.17-3.74)	0.569
Ca (mmol/L)	2.35±0.11	2.36±0.11	2.34±0.11	0.146
P (mmol/L)	1.25±0.19	1.24±0.20	1.27±0.17	0.138
FBG (mmol/L)	8.21±3.09	8.62±3.11	7.79±2.99	0.080
SUA(μmol/L)	326.15±103.13	338.21±104.33	284.23±89.51	<0.001
SCR(μmol/L)	59.90(49.55-70.05)	60.85(51.05-71.95)	52.70(45.60-64.95)	<0.001
PTH (pg/ml)	34.02(26.82-45.20)	33.49(26.55-43.86)	37.18(28.43-48.50)	0.077
N-MID Ost (ng/ml)	11.63(8.84-15.13)	11.01(8.58-14.19)	13.92(10.46-17.47)	<0.001
β-CTX (ng/ml)	0.39(0.26-0.56)	0.36(0.25-0.53)	0.46(0.34-0.62)	<0.001
T-PINP (ng/ml)	40.87(31.17-53.24)	39.74(30.40-49.45)	48.66(36.44-62.51)	<0.001
25-VIT D (ng/ml)	13.80(10.20-18.60)	14.10(10.60-18.70)	12.45(9.30-16.96)	0.019
WBC(10ˆ^12^/L)	6.23±1.78	6.28±1.81	6.07±1.65	0.246
HGB (g/L)	138.67±16.99	139.97±16.46	133.63±18.09	<0.001
Lymph(10ˆ^9^/L)	1.94(1.50-2.35)	1.92(1.52-2.32)	1.96(1.46-2.46)	0.551
Mono(10ˆ^9^/L)	0.39(0.31-0.48)	0.40(0.31-0.49)	0.37(0.30-0.45)	0.058
Neut(10ˆ^9^/L)	3.50(2.67-4.37)	3.50(2.74-4.40)	3.42(2.47-4.12)	0.132
FN BMD (g/cm^2^)	0.76±0.14	0.79±0.12	0.61±0.10	<0.001
TH BMD (g/cm^2^)	0.89±0.14	0.95±0.14	0.75±0.10	<0.001
LS BMD (g/cm^2^)	0.94±0.15	0.99±0.13	0.75±0.09	<0.001
FRAX MOF (%)	2.4(1.6-4.13)	2.10(1.5-3.4)	4.86(3.6-6.4)	<0.001
FRAX HF (%)	0.4(0.1-0.83)	0.2(0.1-0.6)	1.2(0.6-2.55)	<0.001
Current smoker, n (%)	163(25.5%)	140(27.6%)	23(17.6%)	0.019
Drinking habits, n (%)	193(30.2%)	165(32.5%)	28(21.4%)	0.014
Coronary heart disease, n (%)	155(24.2%)	108(21.2%)	47(35.9%)	<0.001
Hypertension, n (%)	314(49.1%)	242(47.5%)	72(55.0%)	0.130

BMI, body mass index; HbA1c, glycated hemoglobin A1C; ALP, Alkaline phosphatase; HDL, high-density lipoprotein cholesterol; LDL, low-density lipoprotein cholesterol; TC, total cholesterol; TG, triglyceride; Ca, calcium; P, phosphorus; FBG, fasting blood glucose; SUA, serum uric acid; SCR, serum creatinine; PTH, parathyroid hormone; N-MID Ost, N-terminal medium molecular fragment of osteocalcin; β-Cros, β-Collagen special sequence; T-PINP, Total type I collagen amino-terminal extension peptide; 25-VIT D, 25-hydroxyvitamin; WBC, white blood cells; HGB, hemoglobin; Lymph, lymphocytes; Mono, monocytes; Neut, neutrophils; BMD, bone mineral density; FN, femoral neck; TH, total hip; LS, lumbar spine; FRAX, fracture risk assessment tool; MOF, major fracture; HF, hip fracture.

a T-Score < -2.5 SD at any site on LS, FN or TH.

### 3.2 Comparison of BMD between normal and anemic groups in different subgroups stratified by age and sex

**Figure 1:**
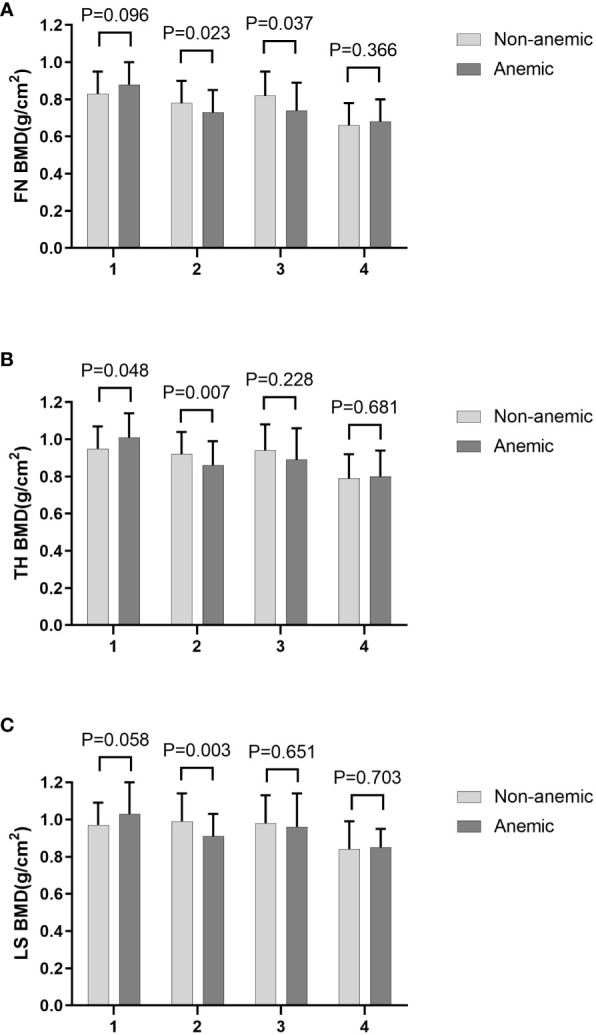
FN BMD **(A)**, TH BMD **(B)** and LS BMD **(C)** according to different anemia status in different subgroups stratified by age and sex. 1. non-older men, 2. older men, 3. non-older women, 4. older women.

Compared to individuals without anemia, participants with anemia had significantly lower FN BMD (**Table 2**). This phenomenon persisted among older men and non-older women (**Table 3**). TH BMD was significantly lower in those with anemia in older men (≥55 years) and higher in anemic non-older men (<55 years). This was not the case in female patients.LS BMD was significantly lower in older men (**Figure 1**). These findings suggest that the effects of HGB levels on bone health in patients with T2DM may be influenced by age and sex. Furthermore, a notable distinction in HGB levels between the older and non-older male groups was observed, whereas no such distinction was observed among females (**Table 4**).

**Table 2:** General characteristics of the participants and comparison of characteristics grouped according to anemia.

Variables	Total (n=641)	Non-anemic (n=535)	Anemic (n=106)	P
Age (years)	56.03±13.35	55.24±13.60	59.55±11.64	<0.001
BMI (kg/m^2^)	25.90±3.98	25.94±3.88	25.57±4.45	0.385
Diabetes duration (years)	10(2-16)	8.00(2-15)	11.5(5-20)	0.001
HbA1c (%)	9.12±2.12	9.15±2.11	8.93±2.29	0.336
ALP (U/L)	76(61.5-92)	76(62-93)	71(60.25-89.75)	0.253
TG (mmol/L)	1.43(0.95-2.09)	1.46(0.99-2.15)	1.23(0.77-1.86)	0.004
TC (mmol/L)	4.85±1.32	4.90±1.27	4.58±1.52	0.020
HDL (mmol/L)	1.19(1.00-1.44)	1.18(0.99-1.44)	1.25(1.05-1.53)	0.091
LDL (mmol/L)	3.02(2.36-3.67)	3.07(2.45-3.69)	2.76(1.93-3.63)	0.002
Ca (mmol/L)	2.35±0.11	2.36±0.10	2.30±0.13	<0.001
P (mmol/L)	1.25±0.19	1.24±0.19	1.29±0.22	0.020
HBG (g/L)	138.67±16.99	143.81±12.69	112.72±11.17	<0.001
FN BMD (g/cm^2^)	0.76±0.14	0.77±0.14	0.74±0.14	0.049
TH BMD (g/cm^2^)	0.89±0.14	0.90±0.14	0.87±0.15	0.086
LS BMD (g/cm^2^)	0.94±0.15	0.94±0.15	0.92±0.16	0.179
FRAX MOF (%)	2.4(1.6-4.1)	2.3(1.6-4.0)	3.0(1.7-4.8)	0.012
FRAX HF (%)	0.4(0.1-0.8)	0.3(0.1-0.8)	0.5(0.1-1.1)	0.073

**Table 3:** Comparison of FN BMD in different subgroups grouped according to anemia.

	Non-anemic (n=535)	Anemic (n=106)	P
Total	0.77±0.14	0.74±0.14	0.049
<55years	0.82±0.12	0.82±0.15	0.753
≥55years	0.72±0.13	0.70±0.12	0.180
Male	0.80±0.12	0.76±0.14	0.151
<55years	0.83±0.12	0.88±0.12	0.077
≥55years	0.78±0.12	0.73±0.12	0.014
Female	0.71±0.14	0.70±0.13	0.388
<55years	0.82±0.13	0.74±0.15	0.049
≥55years	0.66±0.12	0.68±0.12	0.538

**Table 4:** Comparison of HGB among different subgroups.

	Total	<55years	≥55years	P
Total	138.67±16.99	142.80±18.21	135.89±15.53	<0.001
Male	144.73±16.13	148.85±16.53 (n=88)	141.25±14.96 (n=182)	<0.001
Female	130.34±14.44	131.11±15.46 (n=171)	129.97±13.95 (n=201)	0.542
P	<0.001	<0.001	<0.001	

### 3.3 Associations among HGB level and BMD and FRAX 10-year probability of MOFs and HFs in different subgroups stratified by age and sex

Spearman’s correlation analysis was performed to examine the associations between HGB levels and BMD and the FRAX 10-year probability of MOFs and HFs. As indicated in **Table 5**, within the T2DM population, a positive correlation was observed between HGB levels and BMD at all sites, whereas a negative correlation was observed between HGB levels and FRAX 10-year probability of MOF and HF. In male patients, a positive correlation was found between HGB levels and FN and TH BMD in the older population (≥55 years), whereas in the non-older population (<55 years), no correlation was observed between HGB level and BMD, MOF, and HF. Among female patients, HGB levels were positively associated with BMD at all sites and negatively associated with FRAX 10-year probability of MOF and HF in the non-older population (<55 years). However, this association was not observed in the older population (≥55 years).

**Table 5:** Pearson and partial correlation analyses between HGB and BMDs and 10-year probability of MOF and HF in patients with T2DM.

	FN BMD		TH BMD		LS BMD		FRAX MOF (%)		FRAX HF (%)	
	r	P	r	P	r	P	r	P	r	P
Total	0.248	<0.001	0.230	<0.001	0.168	<0.001	-0.253	<0.001	-0.145	<0.001
Male	0.134	0.010	0.114	0.028	0.007	0.898	-0.189	<0.001	-0.126	0.017
<55years	-0.026	0.739	-0.041	0.598	-0.102	0.186	-0.018	0.823	0.044	0.588
≥55years	0.179	0.011	0.180	0.010	0.100	0.161	-0.120	0.092	-0.103	0.146
Female	0.139	0.022	0.110	0.071	0.104	0.088	-0.048	0.434	-0.025	0.682
<55years	0.293	0.006	0.224	0.036	0.214	0.046	-0.292	0.008	-0.266	0.016
≥55years	0.041	0.584	0.035	0.644	0.027	0.716	-0.017	0.816	-0.062	0.996

Multiple regression analyses were performed to test the independent effects of HGB level on BMD and the 10-year probability of fracture risk in different subgroups. In older men, HGB level was an independent determinant of BMD and positively correlated with FN and TH BMD, but not in non-older men with T2DM (**Table 6**). In non-older women, HGB level was an independent determinant of BMD and fracture risk, positively associated with BMD, and negatively associated with the 10-year probability of MOF and HF. However, this correlation was not observed in older women with T2DM.

**Table 6:** Multivariate linear regression analysis of the association between HGB and BMDs and 10-year probability of MOF and HF in patients with T2DM.

	FN BMD		TH BMD		LS BMD		FRAX MOF (%)		FRAX HF (%)	
	β	P	β	P	β	P	β	P	β	P
Male										
<55years	0.002	0.983	0.020	0.817	-0.032	0.713	-0.090	0.345	-0.058	0.539
≥55years	0.199	0.008	0.206	0.005	0.141	0.058	-0.147	0.053	-0.129	0.087
Female										
<55years	0.297	0.006	0.251	0.026	0.262	0.020	-0.317	0.006	-0.276	0.022
≥55years	0.076	0.371	0.079	0.348	0.052	0.525	-0.058	0.494	-0.045	0.590

Adjusted for ALP; HDL; HbA1c; SCR; 25-VIT D.

### 3.4 GAMs with HGB level on BMD and FRAX 10-year probability of MOF and HF

**Figure 2:**
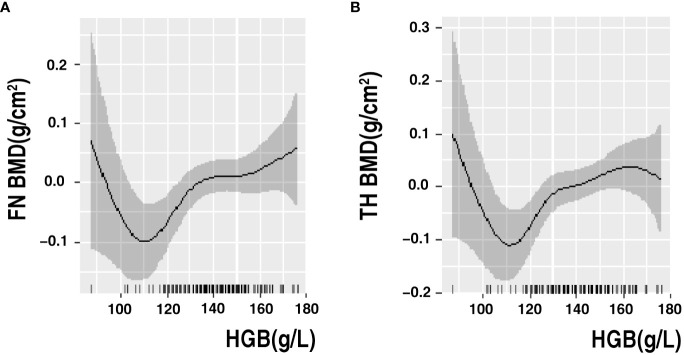
GAMs analysis of HGB with FN BMD **(A)** and TH BMD **(B)** in older men. The solid black line represents the smooth curve fit between variables. Gray bands represent the 95% confidence interval from the fit. Adjusted for ALP, HDL, HbA1c, SCR and 25-VIT D.

**Figure 3:**
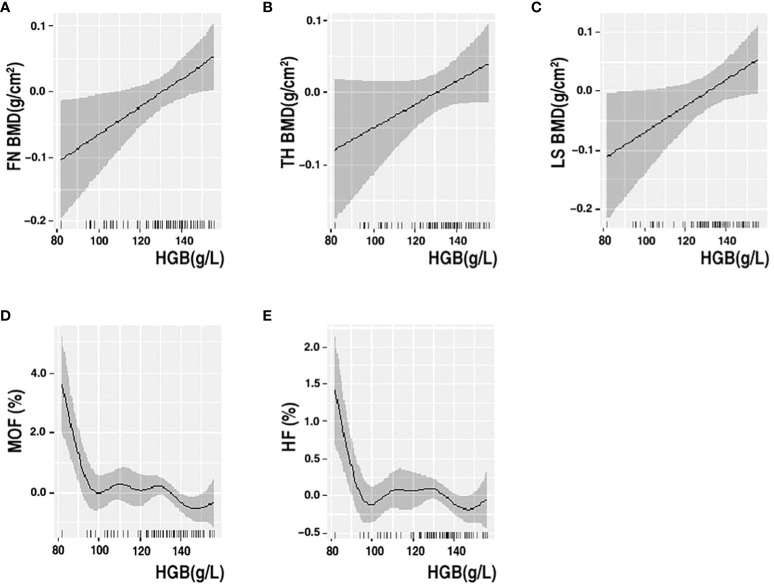
GAMs analysis of HGB with FN BMD **(A)**, TH BMD **(B)**, LS BMD **(C)**, MOF **(D)** and HF **(E)** in non-older women. The solid black line represents the smooth curve fit between variables. Gray bands represent the 95% confidence interval from the fit. Adjusted for ALP, HDL, HbA1c, SCR and 25-VIT D.

Multiple linear regression analysis revealed a linear association between HGB levels and BMD in older men and non-older women with T2DM. We further performed GAMs of the association between HGB level, BMD, and fracture risk to determine their exact correlations in the subgroups. The results of the GAMs analyses showed that the association between HGB level and BMD varied across subgroups. The association between HGB level and FN BMD was linearly negative when HGB was <115 g/L and approximately linearly positive when HGB was >115 g/L in older male patients. The association between HGB level and TH BMD was linearly negative when HGB was <115 g/L, approximately positive when HGB level was between 115 g/L and 170 g/L and became relatively negative when HGB was >170 g/L in older male patients (**Figure 2**). In non-older female patients with T2DM, the association between HGB level and BMD was linearly positive, and the association between HGB level, MOF, and HF showed a non-linear correlation with MOF and HF (**Figure 3**).

## 4 Discussion

In our study, HGB levels were lower in patients with osteoporosis than in controls, FN BMD were lower in patients with anemia than in those without anemia, and interestingly, only in older men or non-older women. In older men, HGB level was an independent determinant of BMD and was positively correlated with FN and TH BMD. In non-older women, HGB level was an independent determinant of BMD and fracture risk, positively associated with BMDs and negatively associated with 10-year probability of fracture risk. Meanwhile, GAMs revealed a positive linear association between HGB level and BMDs in non-older female patients but not in older male patients.

Numerous clinical studies have shown that HGB level has a protective effect on the bone in the general population. In countries such as China (25), Korea (26), Sweden (27), and Turkey (28), HGB level is significantly and positively associated with BMD, whereas anemia is independently associated with an increased risk of fracture (29). However, a clinical study of postmenopausal Chinese women showed a negative correlation between HGB level and BMD (30). Meanwhile, in a large U.S. study, no association between HGB level and BMD was observed after excluding potential confounders (31). Furthermore, the observed inconsistency could potentially stem from ethnic disparities in the impact of HGB on bone health, as numerous studies have documented variations in bone health among different ethnic groups, attributed to factors such as ethnic background and multifaceted environmental influences encompassing nutrition, cultural practices, socioeconomic circumstances, sunlight exposure, and levels of physical activity, among others (32–36). The potential mechanisms underlying the relationship between HGB level and BMD are as follows: First, relative hypoxia accompanied by reduced HGB levels may adversely affect bone metabolism through direct effects of pO2 and indirect effects on bone-forming and resorbing cells (37, 38). Effects of hypoxia on bone marrow mesenchymal stem cells through stimulation of fibroblast proliferation, increased osteogenic potential, and stimulation of adipogenesis (39). An experimental study has shown that hypoxia results in a three-fold increase in osteoclast formation and a 10-fold increase in resorption pit formation (40). Karul et al. reported an independent association between illness severity and BMD parameters in patients with chronic obstructive pulmonary disease (41). In addition, the associated oxidative stress due to reduced HGB levels may directly interfere with bone mass and bone metabolism (42, 43). Second, inflammation may mediate the association between HGB levels and bone metabolism. Studies have shown that pro-inflammatory cytokines affect hematopoiesis (44). Finally, another possible mechanism by which anemia affects the bones is elevated blood erythropoietin (EPO) levels. EPO controls erythrocyte proliferation and differentiation (45). Several preclinical studies have shown that EPO has a detrimental effect on bone mass in adult rodents; however, other studies in growing mice using trauma models have reported the stimulation of bone formation by EPO (46).

Anemia is highly prevalent in individuals with T2DM, with a reported prevalence range of 20%–44% in various populations (47, 48). However, there is no consensus on the association between HGB levels, diabetes, and osteoporosis. In the present study, we found that HGB levels were lower in patients with T2DM combined with osteoporosis than in controls, which is partially consistent with the findings of Xiu et al., who found that HGB levels were lower in men with T2DM combined with osteoporosis than in those with normal bone density (49). At the same time, FN BMD was lower in patients with T2DM combined with anemia than in the control group, which is partially consistent with the findings of Fei et al., who found that patients with low HGB levels combined with T2DM had lower BMD and a higher prevalence of osteoporosis (24). At the same time, we found that TH BMD was significantly lower in anemic older men and higher in anemic non-older men. The possible reasons are as follows: (1) Non-older men have more active bone metabolism and higher compensatory function than older men (50), which may explain the slightly higher BMD in the anemic group of non-older men; (2) Non-older men with higher HGB levels than older men are less anemic and have a relatively weaker impact on bone metabolism, resulting in higher BMD in non-older men; (3) The anemic group was smaller than the non-anemic group, so we cannot exclude the possibility of analytical bias. Interestingly, we found for the first time that this difference exists only in older men and non-older women, suggesting that there may be sex- and age-related differences in the effects of HGB levels on bone in the same patient. We further found that HGB level was an independent determinant of BMD and was positively correlated with FN and TH BMD in older men. HGB level was an independent determinant of BMD and fracture risk, being positively associated with BMD and negatively associated with 10-year probability of fracture risk in non-older women. There is a lack of clinical data on the relationship between HGB levels, BMD, and fracture risk in patients with T2DM. One study found a positive correlation between HGB levels and BMD in Chinese patients with T2DM stratified by sex (24). Another study found that higher HGB level was protective against osteoporosis in older men with T2DM stratified by sex but not age (49). In addition, none of the above studies explored the relationship between HGB levels and the 10-year probability of fracture risk. A U.S. cohort study found an increased risk of non-spinal fractures independent of bone density and bone loss over time in older men with anemia (51). Similarly, the MrOS Sweden cohort found that anemia was associated with an increased risk of fractures and non-vertebral osteoporotic fractures (52).

We found for the first time that HGB level was positively correlated with BMD in patients with T2DM and that HGB level was an independent influencer of BMD. Interestingly, this phenomenon exists only in older men and non-older women. In addition, HGB level was negatively associated with 10-year probability of fracture risk and had an independent influence on 10-year probability of fracture risk, which occurred only in non-older women. Explanation of the above phenomenon: (1) For males, it has been previously reported that low HGB level affects bone metabolism through disturbances such as increased osteoclasts from hypoxia (37, 38), oxidative stress (42), and increased EPO (45), and that low HGB levels are associated with lower BMD. Although HGB levels were significantly lower in older men than in non-older men in our study population, we observed similar results. Therefore, there may be dose variability in the effect of HGB levels on bone in the male population, with low HGB levels correlating more significantly with BMD, and several studies support our results (24, 25). Second, testosterone inhibits osteoclast formation in a dose-dependent manner (53). In older men, sexual function declines due to aging (54, 55), and the long-term toxicity of hyperglycemia (56), leading to a dramatic drop in testosterone levels in men, and low testosterone levels attenuate the inhibitory effect of osteoclasts and increased bone resorption, the bone metabolism system begins to oscillate disruptively. Studies have confirmed that testosterone mediates the relationship between HGB levels and bone loss (57), and low testosterone levels are thought to be associated with anemia (low HGB levels) and reduced BMD in men (58, 59), so In patients with T2DM, the HGB-BMD correlation is more pronounced in older men. Finally, testosterone induces an increase in HGB levels by stimulating EPO (60) as well as decreasing ferritin and hepatic phospholipid concentrations, and in older men, low testosterone levels reduce this induction, hence the above phenomenon. (2) For females, first, we found that BMD was significantly lower in the anemic population than in the non-anemic population, a phenomenon that exists only in the middle-aged female population, further supporting our results. Finally, estrogen is known to play a crucial role in maintaining bone mass in women (61), and estrogen changes drastically from perimenopause (around 45 years of age), leading to disturbances in bone metabolism (62). In our cohort, the proportion of women aged 45–55 years accounted for 59.1% of middle-aged women, and estrogen-mediated the effects of HGB levels on bone in middle-aged women (57); therefore, in patients with T2DM, the HGB-BMD correlation was more significant in middle-aged women. In contrast, estrogen levels are extremely low in older female patients, and the effects on the bone are in a resting state; therefore, there is no correlation between HGB levels and BMD. Admittedly, this phenomenon may have other mechanisms or causes.

Linear regression is a parametric analysis technique that incorporates certain assumptions about data. If the data processed using the parametric technique does not conform to its assumptions, the analysis results may be weak or biased. GAMs are nonparametric regressions that relax linearity assumptions and detect patterns that might have been missed by parametric techniques. Various authoritative studies, including those in ecology, biology, and human medicine, are increasingly utilizing this model. Our study employed a novel model to analyze the association between HGB levels and bone health. In non-older female patients with T2DM, the association between HGB levels and BMD was linearly positive, and the association between HGB levels and MOF and HF showed a non-linear correlation with MOF and HF. The association between HGB levels and FN BMD was linearly negative when HGB was <115 g/L and approximately linearly positive when HGB was >115 g/L in older male patients. The association between HGB levels and TH BMD was linearly negative when HGB was <115 g/L, approximately positive when HGB level was between 115 g/L and 170 g/L and became relatively negative when HGB was >170 g/L in older male patients. This is partially similar to the study by Cui et al., who found that the effect of HGB levels on BMD was not a unidirectional linear relationship in men with T2DM and that HGB level was negatively correlated with BMD in the anemic population; however, they did not stratify by age (49). It has been suggested that anemia is a risk factor for osteoporosis and that part of its negative correlation is mediated by testosterone (57).

The current study had several limitations. (1) Because it was a cross-sectional study, it was not possible to determine whether there was a causal relationship between HGB levels and bone health in the T2DM population; (2) We did not adjust for all confounding factors that may affect bone metabolism and HGB levels, such as the timing of menopause, insulin and some antidiabetic medications, physical activity, daily diet, and diabetic complications; (3) A highly heterogeneous group of participants (18–85 years old); (4) We did not collect sufficient data on long-term endpoints such as osteoporotic fractures and hip fractures and used HFs and MOFs as surrogates; (5) This study did not include healthy controls; (6) Due to the lack of measurements of serum erythropoietin, iron, vitamin B12, or folate levels, we were unable to determine the etiology of anemia in these participants. Therefore, these results should be interpreted with caution.

In conclusion, the present study demonstrates for the first time that lower HGB levels are associated with lower FN and TH BMD in an older male T2DM population and that lower HGB levels are associated with lower BMD and higher future fracture risk in a non-older female T2DM population. In addition, relatively low HGB levels may be an independent risk factor for osteoporosis and osteoporotic fractures in this population. Further studies are needed to confirm these results and investigate the potential mechanisms.

## Data availability statement

The original contributions presented in the study are included in the article/**Supplementary Material**. Further inquiries can be directed to the corresponding authors.

## Ethics statement

The studies involving humans were approved by Institutional Review Board of Shandong Provincial Hospital affiliated with Shandong First Medical University. The studies were conducted in accordance with the local legislation and institutional requirements. The participants provided their written informed consent to participate in this study. Written informed consent was obtained from the individual(s) for the publication of any potentially identifiable images or data included in this article.

## Author contributions

RL: Writing – original draft, Data curation, Investigation, Methodology. NX: Writing – original draft. YG: Writing – original draft. YW: Writing – original draft. YL: Writing – original draft. XZ: Writing – original draft. WJ: Writing – original draft. JW: Writing – original draft. XZ: Writing – original draft. LZ: Writing – original draft. LZ: Writing – original draft. YZ: Writing – review & editing. JX: Writing – review & editing.
